# Closed-Form Solution and Experimental Verification for the Axisymmetric Deformation Problem of Blistering Circular Thin Polymer Films under Uniformly Distributed Gas Pressure

**DOI:** 10.3390/polym12051130

**Published:** 2020-05-14

**Authors:** Zhi-Xin Yang, Xiao-Ting He, Si-Rui Wen

**Affiliations:** 1 School of Civil Engineering, Chongqing University, Chongqing 400045, China; 20141602063@cqu.edu.cn (Z.-X.Y.); wensirui123@163.com (S.-R.W.); 2 Key Laboratory of New Technology for Construction of Cities in Mountain Area, Chongqing University, Ministry of Education, Chongqing 400045, China

**Keywords:** thin-film/substrate systems, pressure blister test, uniformly distributed gas pressure, experimental verification, closed-from solution

## Abstract

The existing studies indicate that the measurement formulas used in blister test techniques, which are used to measure the mechanical properties of thin-film/substrate systems, are usually given based on an approximation—that is, the applied direction of the uniformly distributed transverse load is always vertical, while the applied direction of the uniformly distributed gas pressure is always perpendicular to the surface of the thin film. This approximation will lead to a large measurement error. In this study, we obtained the analytical solution to the problem of axisymmetric deformation of blistering circular thin polymer films under the action of uniformly distributed gas pressure via the power series method. An example is given to illustrate the error caused by the approximation mentioned above, and the validity of the solution presented here is verified. The result shows that the chance of error caused by the approximation increases with the increase in the applied load, and it far exceeds the allowable error of measurement when the applied load is relatively large. In addition, the related experiments of the blistering circular thin polymer film under uniformly distributed gas pressure are carried out, and the experimental results are compared with the theoretical results. The comparison results show that the analytical solution given in this paper is correct. The solution presented here is of great significance to improve the measurement accuracy of the blister test technique.

## 1. Introduction

Thin-film/substrate systems have found increasing application in many fields, such as civil engineering, mechanics and biotechnology [1,2,3,4,5,6]. Usually, the reliability of thin-film/substrate systems depends mainly on the mechanical properties of thin-film/substrate systems (including the mechanical properties of surface thin film and interfacial adhesive strength of thin-film/substrates). Therefore, in order to know the reliability of thin-film/substrate systems more accurately, it is necessary to measure the mechanical properties of thin-film/substrate systems precisely. The measurement formula used is usually based on the analytical solution of the corresponding mechanical problem, so it is also necessary to give the analytical solution of the corresponding mechanical problem.

**Figure 1:**
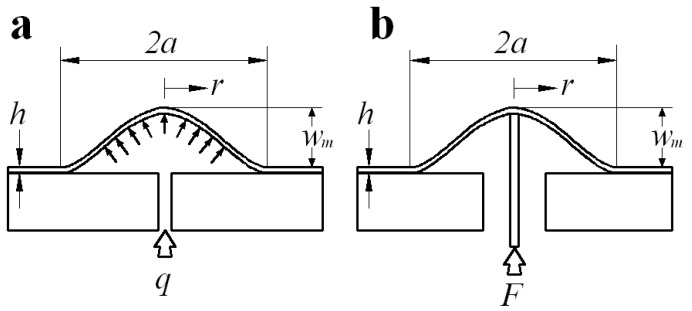
Sketches of the loading configuration of blister tests. (**a**) a pressurized circular blister configuration and (**b**) a shaft-loaded circular blister configuration.

**Figure 2:**
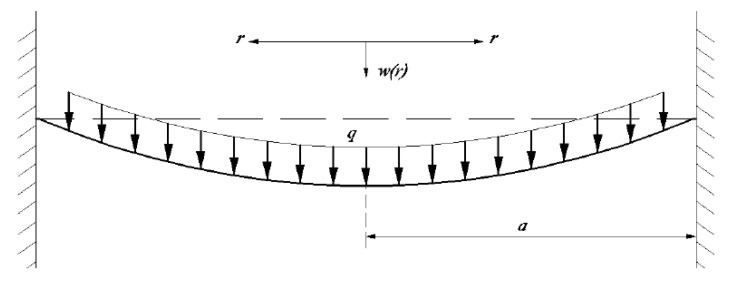
Sketch of circular thin film under uniformly distributed transverse load.

To date, many test techniques have been used for the measurement of the mechanical properties of thin-film/substrate systems [7,8,9,10,11,12,13,14,15,16,17,18], of which the blister testing method is common and realizes the synchronous measurement (the mechanical properties of the thin film and the adhesive strength of the thin-film/substrate systems can be measured simultaneously) [19,20,21]. The blister test technique was first suggested by Dannenberg [22], and was then developed into many variant forms by subsequent investigators [23,24,25,26]. All blister tests can be classified into two major variants according to the loading method: (i) gas pressure loading (corresponding to a pressure blister test, as shown in Figure 1a where *q* denotes applied load, *a* denotes the radius of film, *h* denotes the thickness of film, *r* denotes the radial coordinate and *w_m_* denotes the max transversal displacement of the circular membrane), and (ii) shaft-loading (corresponding to a shaft-loaded blister test, as shown in Figure 1b where *F* denotes applied load). In the pressure blister test, the thin film is pressurized progressively by working gas, until an axisymmetric blister crack runs into the interface of thin-film/substrate systems. From Figure 1a, it can be seen that the delamination process of the thin film from the substrate can be simplified as the mechanical problem of axisymmetric deformation of circular thin film under the action of uniformly distributed gas pressure. The measurement formulas used in the pressure blister test are given based on the analytical solution of the mechanical problem. However, due to the difficulty in obtaining the analytical solution of axisymmetric deformation problem of circular thin film under uniformly distributed gas pressure, the existing measurement formulas are all given by the analytical solution of the axisymmetric deformation problem of circular thin film under uniformly distributed transverse load. The mechanical model of this is shown in Figure 2 [15]. The problem of axisymmetric deformation of circular thin film under uniformly distributed transverse load was originally dealt with by Hencky [27]; therefore, it is widely known as the Hencky problem for short, and its solution is known as the Hencky solution [28].

From Figure 1a and Figure 2, it can be seen that the applied direction of the uniformly distributed transverse load is always vertical, while the applied direction of the uniformly distributed gas pressure is always perpendicular to the surface of the thin film. When the gas pressure is very small, the deformation of the thin film will be correspondingly small. Thus, the component of uniformly distributed gas pressure along the horizontal direction is also relatively small. In this case, the uniformly distributed gas pressure may be approximately replaced by the uniformly distributed transverse load without much error. However, with the increase in uniformly distributed gas pressure, the deformation of the thin film will increase correspondingly, meaning that the component of uniformly distributed gas pressure along the horizontal direction is no longer too small, and will have a great impact on the deformation of the thin film. If the uniformly distributed gas pressure is replaced by the uniformly distributed transverse load, obviously there will be a big error. In addition, thin films are usually made of flexible materials, which can easily produce large deformations under the action of external loads. Therefore, it is obviously inappropriate to replace the uniformly distributed gas pressure with the uniformly distributed transverse load. As a result, in order to obtain an accurate measurement formula of the blister test technique, it is necessary to give an analytical solution of the axisymmetric deformation problem of circular thin film under uniformly distributed gas pressure.

In this paper, the closed-form solution of the problem of axisymmetric deformation of the blistering circular thin film under the action of uniformly distributed gas pressure was presented by the power series method. In Section 2, the governing equations of the problem solved here will be given and dimensionless, and the dimensionless governing equations will be solved by the power series method. The solution presented in this paper will be compared with the well-known Hencky solution [28] in Section 3. Next, in Section 4, we will conduct the related experiments of the blistering circular thin films under uniformly distributed gas pressure and compare the experimental results with the solution presented here. According to the results mentioned above, some main conclusions will be drawn in Section 5. The work presented here is of great significance and aims to improve the measurement accuracy of blister test technique. In addition, thin and ultrathin films are widely used for gas and solvent separation [29,30]. Thus, the work of this paper also has a certain guiding role for gas and solvent separation.

## 2. Membrane Equation and Its Solution

### 2.1. Establishment of Membrane Equations

**Figure 3:**
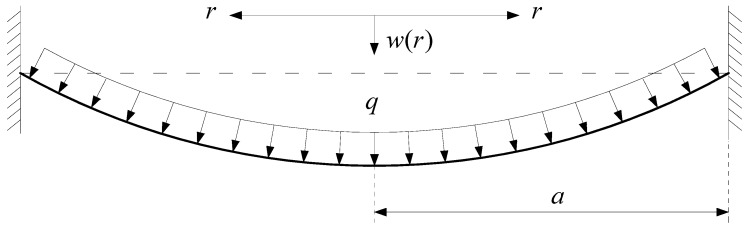
Sketch of circular membrane under uniformly distributed gas pressure.

A uniformly distributed gas pressure, q, is applied onto the surface of a peripherally fixed circular membrane with Young’s modulus of elasticity, *E*, Poisson’s ratio, *v*, thickness, *h*, and radius, *a*, as shown in Figure 3, where the dashed lines denote the initial flat circular membrane, *r* is the radial coordinate and *w* is the transversal displacement of the circular membrane.

**Figure 4:**
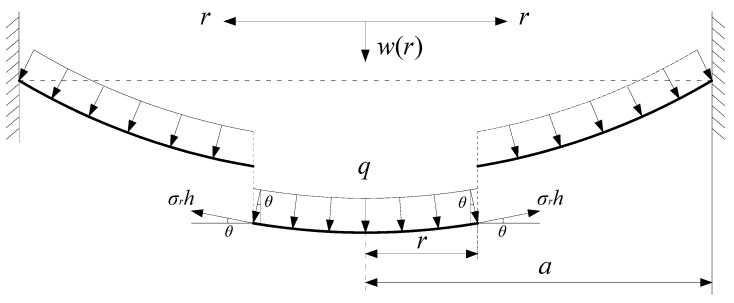
The equilibrium diagram of the central portion (r<a) of the circular membrane.

Let us take a piece of the central portion of the circular membrane, whose radius is 0<r<a, with a view to studying this membrane’s static problem of equilibrium under the action of the uniformly distributed gas pressure q and the membrane force $\sigma_{r}h$ acted on the boundary, as shown in Figure 4, where $\sigma_{r}$ is the radial stress and θ is the sloped angle of the membrane after loading.

**Figure 5:**
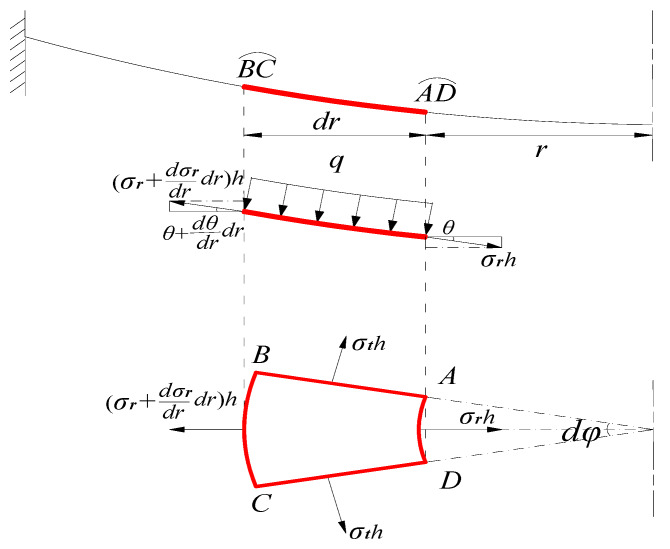
Sketch of the differential element body ABCD where the red curves denotes the Profile of the thin film.

Then, a wedge differential element, ABCD, is cut out from the circular membrane by the two radial sections AB, CD, normal to the membrane, and by two cylindrical sections AD, BC, also normal to the membrane, as shown in Figure 5, in which the normal stress component in the radial direction is denoted by $\sigma_{r}$, another component in the circumferential direction by $\sigma_{t}$ and φ is the other coordinate parameter, i.e., the angular coordinate in the cylindrical coordinate (r,φ,w). There are four normal forces acting on the four sides of this element, in which the radial membrane force acting on the side AD is $\sigma_{r}h$ and the radial membrane force on the side BC is $(\sigma_{r}+\frac{d\sigma_{r}}{dr}dr)h$. The sides AB and CD are subjected to the same circumferential membrane force $\sigma_{t}h$ due to axisymmetric characteristics.

It is easily seen from Figure 5 that the equilibrium equation along the *w*-axis direction is

(1) $$\begin{array}{l} \left(\sigma_{r}+\frac{d\sigma_{r}}{dr}dr\right)h\left(r+dr\right)d\varphi \sin\left(\theta +\frac{d\theta }{dr}dr\right)\\ -\sigma_{r}hrd\varphi \sin\theta =q\frac{1}{2}\frac{\left[{\left(r+dr\right)}^{2}-r^{2}\right]}{\cos\theta }d\varphi \cos\theta \end{array}.$$

By summing up the components of forces along the *r*-axis direction, we can obtain the equilibrium equation,

(2) $$\begin{array}{l} \left(\sigma_{r}+\frac{d\sigma_{r}}{dr}dr\right)h\left(r+dr\right)d\varphi \cos\left(\theta +\frac{d\theta }{dr}dr\right)-\sigma_{r}hrd\varphi \cos\theta \\ -2\sigma_{t}h\frac{dr}{\cos\theta }\sin\left(\frac{d\varphi }{2}\right)+q\frac{1}{2}\frac{\left[{\left(r+dr\right)}^{2}-r^{2}\right]}{\cos\theta }d\varphi \sin\theta =0 \end{array},$$

where the body force of the membrane is ignored.

From Equations (1) and (2), we can obtain

(3) $$2\sigma_{r}rh\sin\theta =qr^{2},$$

and

(4) $$h\frac{d\left(\sigma_{r}r\cos\theta \right)}{dr}\cos\theta -\sigma_{t}h+qr\sin\theta =0,$$

where

(5a) $$\sin\theta ≅\tan\theta =-\frac{dw}{dr}$$

and

(5b) $$\cos\theta =\frac{1}{\sqrt{1+\tan^{2}\theta }}=\frac{1}{\sqrt{1+{\left(-dw/dr\right)}^{2}}}.$$

Substituting Equations (5a) and (5b) into Equations (3) and (4), it can be found that

(6) $$-2\sigma_{r}h\frac{dw}{dr}=qr$$

and

(7) $$\sigma_{t}h=h\frac{d\left(\sigma_{r}r/\sqrt{1+{\left(dw/dr\right)}^{2}}\right)}{dr}\frac{1}{\sqrt{1+{\left(dw/dr\right)}^{2}}}-qr\frac{dw}{dr}.$$

The relations of the strain and displacement of the large deflection problem—that is, the so-called geometric equations—still follow the classical geometric equations [31],

(8) $$\begin{array}{l} e_{r}=\frac{du}{dr}+\frac{1}{2}{\left(\frac{dw}{dr}\right)}^{2}\\ e_{t}=\frac{u}{r} \end{array}\},$$

in which $e_{r}$ is the radial strain, $e_{t}$ is the circumferential strain, and u is the radial displacement. The relations of the stress and strain—that is, the so-called physical equations—also follow the classical physical equations [31],

(9) $$\begin{array}{l} \sigma_{r}=\frac{E}{1-\nu^{2}}\left(e_{r}+\nu e_{t}\right)\\ \sigma_{t}=\frac{E}{1-\nu^{2}}\left(e_{t}+\nu e_{r}\right) \end{array}\}.$$

Substituting Equation (8) into Equation (9), we may obtain

(10a) $$h\sigma_{r}=\frac{Eh}{1-\nu^{2}}\left[\frac{du}{dr}+\frac{1}{2}{\left(\frac{dw}{dr}\right)}^{2}+v\frac{u}{r}\right]$$

and

(10b) $$h\sigma_{t}=\frac{Eh}{1-\nu^{2}}\left[\frac{u}{r}+\frac{v}{2}{\left(\frac{dw}{dr}\right)}^{2}+v\frac{du}{dr}\right].$$

By means of Equations (10a), (10b) and (4), we may obtain

(11) $$\frac{u}{r}=\frac{1}{Eh}\left[h\frac{\frac{d\sigma_{r}}{dr}r}{1+{\left(dw/dr\right)}^{2}}+h\frac{\sigma_{r}}{1+{\left(dw/dr\right)}^{2}}-h\frac{\sigma_{r}r\frac{dw}{dr}\frac{d^{2}w}{dr^{2}}}{{\left[1+{\left(dw/dr\right)}^{2}\right]}^{2}}-qr\frac{dw}{dr}-\nu h\sigma_{r}\right].$$

Then substituting the u of Equation (11) into Equation (10a), we obtain

(12) $$\begin{array}{l} h\sigma_{r}=\frac{3h\frac{d\sigma_{r}}{dr}r}{1+{\left(dw/dr\right)}^{2}}+\frac{h\sigma_{r}}{1+{\left(dw/dr\right)}^{2}}-\frac{4h\sigma_{r}r\frac{dw}{dr}\frac{d^{2}w}{dr^{2}}}{{\left[1+{\left(dw/dr\right)}^{2}\right]}^{2}}-2qr\frac{dw}{dr}-\nu h\sigma_{r}\\ +\frac{h\frac{d^{2}\sigma_{r}}{dr^{2}}r^{2}}{1+{\left(dw/dr\right)}^{2}}-\frac{3h\frac{d\sigma_{r}}{dr}r^{2}\frac{dw}{dr}\frac{d^{2}w}{dr^{2}}}{{\left[1+{\left(dw/dr\right)}^{2}\right]}^{2}}-\frac{h\sigma_{r}r^{2}\frac{dw}{dr}\frac{d^{3}w}{dr^{3}}}{{\left[1+{\left(dw/dr\right)}^{2}\right]}^{2}}-\frac{h\sigma_{r}r^{2}{\left(\frac{d^{2}w}{dr^{2}}\right)}^{2}}{{\left[1+{\left(dw/dr\right)}^{2}\right]}^{2}}\\ +\frac{4h\sigma_{r}r^{2}{\left(\frac{dw}{dr}\right)}^{2}{\left(\frac{d^{2}w}{dr^{2}}\right)}^{2}}{{\left[1+{\left(dw/dr\right)}^{2}\right]}^{3}}-qr^{2}\frac{d^{2}w}{dr^{2}}-\nu hr\frac{d\sigma_{r}}{dr}+\frac{Eh}{2}{\left(\frac{dw}{dr}\right)}^{2}+\frac{vh\frac{d\sigma_{r}}{dr}r}{1+{\left(dw/dr\right)}^{2}}\\ +\frac{vh\sigma_{r}}{1+{\left(dw/dr\right)}^{2}}-\frac{vh\sigma_{r}r\frac{dw}{dr}\frac{d^{2}w}{dr^{2}}}{{\left[1+{\left(dw/dr\right)}^{2}\right]}^{2}}-vqr\frac{dw}{dr} \end{array}.$$

The boundary conditions, under which Equations (6), (7) and (12) can be solved, are

(13a) $$\frac{dw}{dr}=0\;\mathrm{at}\;r=0$$

and

(13b) $$\frac{u}{r}=0\;\mathrm{and}\;w=0\;\mathrm{at}\;r=a.$$

Equations (6), (7) and (12) are three differential equations for the solutions of $\sigma_{r}$, $\sigma_{t}$ and w, which can be solved by the boundary conditions, Equations (13a) and (13b).

### 2.2. Nondimensionalization

Let us introduce the following dimensionless variables

(14) $$Q=\frac{aq}{hE},\;W=\frac{w}{a},\;S_{r}=\frac{\sigma_{r}}{E},\;S_{t}=\frac{\sigma_{t}}{E},\;x=\frac{r}{a},$$

and transform Equations (6), (7), (12) and (11) into

(15) $$Qx+2S_{r}\frac{dW}{dx}=0,$$

(16) $$S_{t}=\frac{\frac{dS_{r}}{dx}x}{1+{\left(dW/dx\right)}^{2}}+\frac{S_{r}}{1+{\left(dW/dx\right)}^{2}}-\frac{S_{r}x\frac{dW}{dx}\frac{d^{2}W}{dx^{2}}}{{\left[1+{\left(dW/dx\right)}^{2}\right]}^{2}}-Qx\frac{dW}{dx},$$

(17) $$\begin{array}{l} \frac{3\frac{dS_{r}}{dx}x}{1+{\left(dW/dx\right)}^{2}}+\frac{S_{r}}{1+{\left(dW/dx\right)}^{2}}-\frac{4S_{r}x\frac{dW}{dx}\frac{d^{2}W}{dx^{2}}}{{\left[1+{\left(dW/dx\right)}^{2}\right]}^{2}}-2Qx\frac{dW}{dx}-\nu S_{r}\\ +\frac{\frac{d^{2}S_{r}}{dx^{2}}x^{2}}{1+{\left(dW/dx\right)}^{2}}-\frac{3\frac{dS_{r}}{dx}x^{2}\frac{dW}{dx}\frac{d^{2}W}{dx^{2}}}{{\left[1+{\left(dW/dx\right)}^{2}\right]}^{2}}-\frac{S_{r}x^{2}\frac{dW}{dx}\frac{d^{3}W}{dx^{3}}}{{\left[1+{\left(dW/dx\right)}^{2}\right]}^{2}}-\frac{S_{r}x^{2}{\left(\frac{d^{2}W}{dx^{2}}\right)}^{2}}{{\left[1+{\left(dW/dx\right)}^{2}\right]}^{2}}\\ +\frac{4S_{r}x^{2}{\left(\frac{dW}{dx}\right)}^{2}{\left(\frac{d^{2}W}{dx^{2}}\right)}^{2}}{{\left[1+{\left(dW/dx\right)}^{2}\right]}^{3}}-Qx^{2}\frac{d^{2}W}{dx^{2}}-\nu x\frac{dS_{r}}{dx}+\frac{1}{2}{\left(\frac{dW}{dx}\right)}^{2}+\frac{v\frac{dS_{r}}{dx}x}{1+{\left(dW/dx\right)}^{2}}\\ +\frac{vS_{r}}{1+{\left(dW/dx\right)}^{2}}-\frac{vS_{r}x\frac{dW}{dx}\frac{d^{2}W}{dx^{2}}}{{\left[1+{\left(dW/dx\right)}^{2}\right]}^{2}}-vQx\frac{dW}{dx}-S_{r}=0 \end{array}$$

and

(18) $$\begin{array}{l} \frac{u}{r}=\frac{1}{{\left[1+{\left(dW/dx\right)}^{2}\right]}^{2}}\{\frac{dS_{r}}{dx}x\left[1+{\left(dW/dx\right)}^{2}\right]+S_{r}\left[1+{\left(dW/dx\right)}^{2}\right]\\ -S_{r}x\frac{dW}{dx}\frac{d^{2}W}{dx^{2}}-Qx\frac{dW}{dx}{\left[1+{\left(dW/dx\right)}^{2}\right]}^{2}-\nu S_{r}{\left[1+{\left(dW/dx\right)}^{2}\right]}^{2}\} \end{array}.$$

Accordingly, the boundary conditions can be transformed into

(19a) $$\frac{dW}{dx}=0\;\mathrm{at}\;x=0$$

and

(19b) $$\begin{array}{l} \frac{dS_{r}}{dx}x\left[1+{\left(dW/dx\right)}^{2}\right]+S_{r}\left[1+{\left(dW/dx\right)}^{2}\right]-S_{r}x\frac{dW}{dx}\frac{d^{2}W}{dx^{2}}\\ -Qx\frac{dW}{dx}{\left[1+{\left(dW/dx\right)}^{2}\right]}^{2}-\nu S_{r}{\left[1+{\left(dW/dx\right)}^{2}\right]}^{2}=0 \end{array}\;\mathrm{and}\;W=0\;\mathrm{at}\;x=1$$

### 2.3. Power Series Solution

Equations (6), (7) and (12) are three differential equations that are usually difficult to solve. Here, we use the power series method to solve them. Note that the radial stress and the transverse displacement are continuous functions; both of them can be expanded in terms of the power series. So the dimensionless radial stress $S_{r}$ and transversal displacement W are simultaneously expanded in the form of power series with respect to x, i.e., let

(20) $$S_{r}\left(x\right)=\sum_{n=0}^{\infty }b_{n}x^{n}$$

and

(21) $$W\left(x\right)=\sum_{n=0}^{\infty }c_{n}x^{n}.$$

After substituting Equations (20), (21) into Equations (15) and (17), the equations are represented by the undetermined constants $b_{n}$ and $c_{n}$. In order to let the expressions on the left-hand of Equations (15) and (17) constantly be zero, the coefficients of all items of $x^{n}$ should be zero. Thus, we can obtain the expressions of the dimensionless stress and transversal displacement with the unknown constants $b_{0}$ and $c_{0}$, such that,

(22) $$\begin{array}{l} S_{r}=b_{0}-\frac{Q^{2}}{64b_{0}^{2}}x^{2}-\frac{Q^{4}}{6144b_{0}^{5}}\left(48\nu b_{0}^{2}+240b_{0}^{2}+1\right)x^{4}-\frac{Q^{6}}{4718592b_{0}^{8}}(1248\nu b_{0}^{2}\\ -6144\nu b_{0}^{3}-43008b_{0}^{3}+8352b_{0}^{2}+13)x^{6}-\frac{Q^{8}}{1509949440b_{0}^{11}}(119808\nu^{2}b_{0}^{4}\\ +2045952\nu b_{0}^{4}-116736\nu b_{0}^{3}+8709120b_{0}^{4}+12960\nu b_{0}^{2}-1038336b_{0}^{3}\\ +106848b_{0}^{2}+85)x^{8}-\frac{Q^{10}}{724775731200b_{0}^{14}}(4520448\nu^{2}b_{0}^{4}-28016640\nu^{2}b_{0}^{5}\\ -498401280\nu b_{0}^{5}+89275392\nu b_{0}^{4}-2092400640b_{0}^{5}-2574336\nu b_{0}^{3}\\ +440732160b_{0}^{4}+199920\nu b_{0}^{2}-27703296b_{0}^{3}+1944528b_{0}^{2}+925)x^{10}-\ldots \end{array}$$

and

(23) $$\begin{array}{l} W=c_{0}-\frac{1}{4}\frac{Q}{b_{0}}x^{2}+\frac{1}{512}\frac{Q^{3}}{b_{0}^{4}}x^{4}-\frac{1}{147456}\frac{Q^{5}}{b_{0}^{7}}\left(96\nu b_{0}^{2}+480b_{0}^{2}+5\right)x^{6}\\ +\frac{Q^{7}}{75497472b_{0}^{10}}\left(43008b_{0}^{3}+6144\nu b_{0}^{3}-14112b_{0}^{2}-2400\nu b_{0}^{2}-55\right)x^{8}\\ -\frac{Q^{9}}{724775731200b_{0}^{13}}(5087232\nu^{2}b_{0}^{4}+71221248\nu b_{0}^{4}+264314880b_{0}^{4}\\ -4276224\nu b_{0}^{3}-35241984b_{0}^{3}+910080\nu b_{0}^{2}+6066432b_{0}^{2}+12600)x^{10}+\ldots \end{array}.$$

It can be seen that Equation (19a) is automatically satisfied by taking the first derivative with respect to x in Equation (23). For the given problem where a, h, E, ν, and q are known in advance, the remaining undetermined constant $b_{0}$ can be determined by substituting Equations (22) and (23) into Equation (19b), and with this known constant $b_{0}$, the undetermined constant $c_{0}$ can be determined by substituting Equation (23) into Equation (19b). As for $S_{t}$, it is easily obtained by direct substitution, so there is no need to illustrate in detail. Thus, the radial stress and transverse displacement of the circular membrane under uniformly distributed gas pressure are determined.

## 3. Results and Discussion

**Figure 6:**
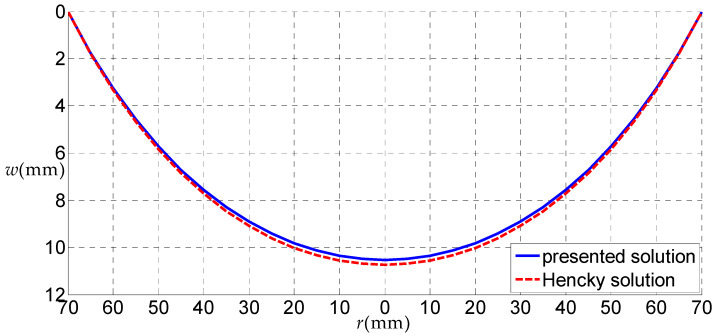
The variations of *w* w with r when q = 0.01 MPa.

**Figure 7:**
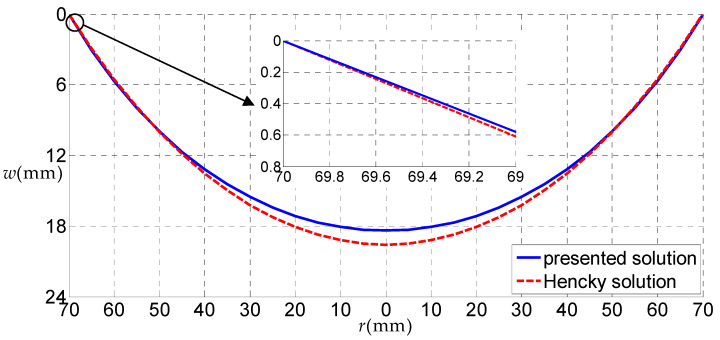
The variations of w with r when q = 0.05 MPa.

**Figure 8:**
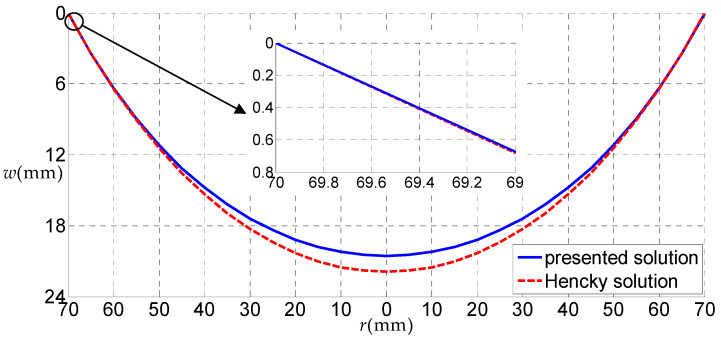
The variations of w with r when q = 0.07 MPa.

**Figure 9:**
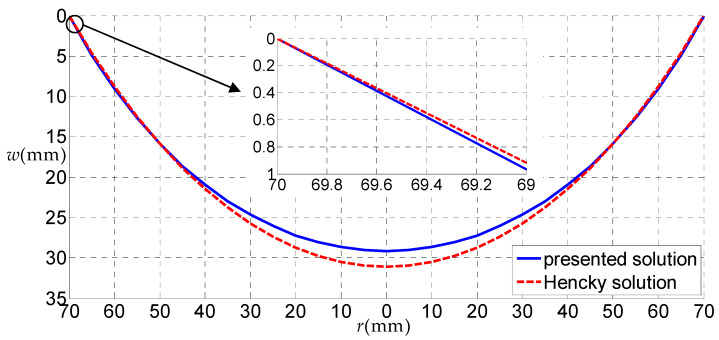
The variations of w with r when q = 0.2 MPa.

**Figure 10:**
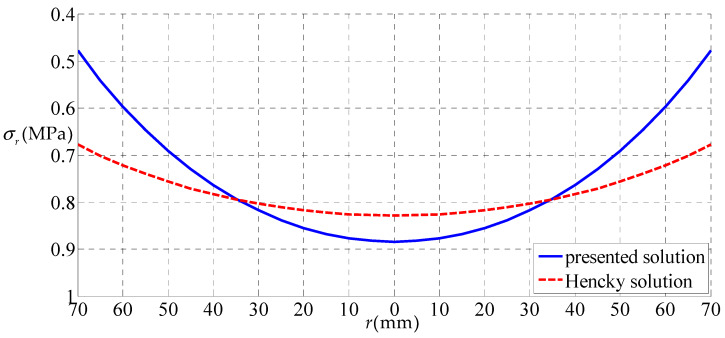
The variations of $\sigma_{r}$ with r when q = 0.07MPa.

Let us consider a rubber circular thin film with a=70 mm, h=5 mm, E=6.11 MPa, ν=0.49 subjected to the uniformly distributed gas pressure q, as a numerical example, to discuss some related issues. Figure 6, Figure 7, Figure 8 and Figure 9 show the variations of w with r when q takes 0.01, 0.05, 0.07 and 0.2 MPa, respectively, and Figure 10 shows the variations of $\sigma_{r}$ with r when q takes 0.07 MPa, where the solid line represents the result obtained by the solution presented here, and the dashed line by the Hencky solution [28].

Theoretically, when the uniformly distributed gas pressure is very small, correspondingly, the deformation of the thin film will also be very small. In this case, the uniformly distributed gas pressure can be approximately regarded as the uniformly distributed transverse load, due to the fact that the horizontal component of the uniformly distributed gas pressure is not obvious. Therefore, when the load is very small, the deflections of the uniformly distributed gas pressure problem and uniformly distributed transverse load problem should be very close. From Figure 6, it can be seen that, when q=0.01 MPa (very small), the solid line is very close to the dash line, which demonstrates, from the side, the validity of the solution presented here.

From Figure 7, Figure 8 and Figure 9, it can be seen that, when q=0.05 MPa, the transverse deflections w(r) obtained by the solution presented here were all smaller than that of the Hencky solution within the entire definition domain. When q=0.07 MPa, the transverse deflection w(r) obtained by the solution presented here was approximately equal to that by Hencky solution in the peripheral region of the circular film, and was smaller than that of the Hencky solution in the central region. Finally, when q increases to 0.2 MPa, the transverse deflection w(r) obtained by the solution presented here was larger than that of the Hencky solution in the peripheral region of the circular film and was smaller than that of the Hencky solution in the central region. Through the comparative analysis of the three cases, it can be seen that when the load was small, the horizontal component of the uniformly distributed gas pressure had little effect on the deflection of the circular thin film, and the uniformly distributed gas pressure can be approximately equivalent to the uniformly distributed transverse load. When the uniformly distributed gas pressure became larger, the horizontal component of the uniformly distributed gas pressure had a greater impact on the deflection of the circular thin film, which is mainly reflected in the outer part of the circular thin film. The horizontal force of the uniformly distributed gas pressure made the outer part of the circular thin film expand horizontally. At this point, if the uniformly distributed gas pressure was replaced by the uniformly distributed transverse load, a large error would have been generated.

From Figure 10, it can be seen that the variation trend of $\sigma_{r}$ obtained by the presented solution and Hencky solution is basically consistent. The $\sigma_{r}$ obtained by the presented solution is always less than that of the Hencky solution on the outer side of the circular film. In the center part of the circular thin film, the $\sigma_{r}$ obtained by the presented solution is all greater than that that of the Hencky solution. It can be seen that both ends of the circular thin film expanded outward due to the horizontal force of uniformly distributed gas pressure. This horizontal component made the circular thin film relaxed and the stress decreased in the outer part, and the circular thin film tightened and the stress increased in the central part.

The measuring formulas used in existing blister test techniques are usually calculated based on the ratio of deflection at  r=a/2 of the thin film to deflection at the center of the thin film. Therefore, Table 1 shows $w_{0}$ and $w_{a/2}$ obtained from the two solutions (the Hencky solution and the solution presented here) and the ratio between them, and also gives the relative error of $w_{0.5a}/w_{0}$. From Table 1, it can be seen that when the gas pressure is small, the error is relatively small, but with the increase in gas pressure, the error gradually increases. Generally, the allowable error of the measurement is 3%, but the error exceeds this allowable value when the load is just 0.2 MPa. When the load is equal to 1.5 MPa, the error is as high as 10.301%. This fully illustrates the necessity and importance of obtaining the analytical solution of axisymmetric deformation of circular thin films under uniformly distributed gas pressure.

## 4. Experimental Analysis

**Figure 11:**
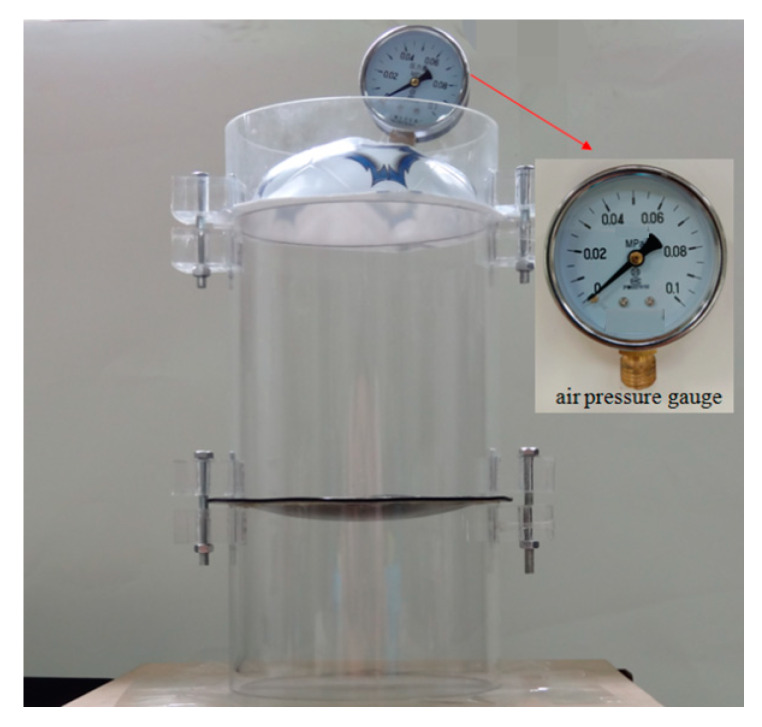
The scheme of the cylinder device.

**Figure 12:**
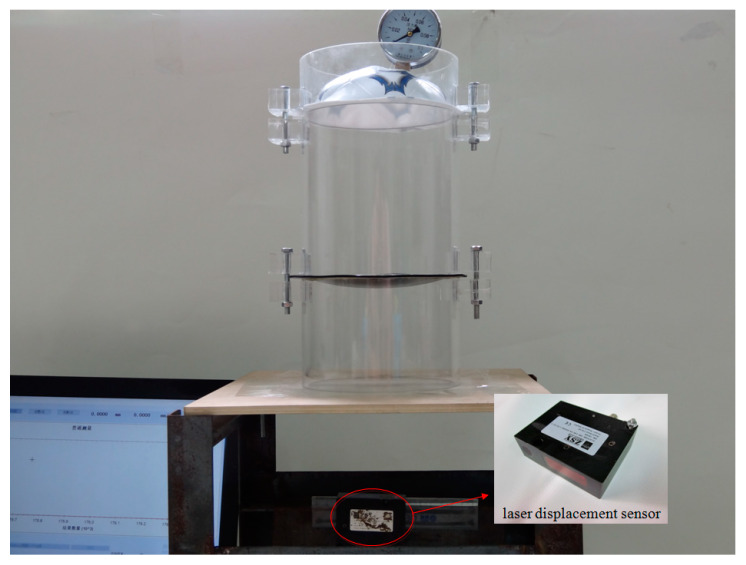
The integral measuring device.

An experiment was conducted to verify the validity of the closed-form solution given in this paper. A rubber film with  h=5 mm,  E=6.11 MPa,  ν=0.49 was clamped by two plastic-steel cylinders with an inner radius of 70 mm and an outer radius of 75 mm. A total of thirteen measuring points were marked every 10mm on the axis of the rubber film, then the other film with the inflation hole and the air pressure gauge was clamped on the upper plastic-steel cylinder. The scheme of cylinder device is shown in Figure 11. The gas pressure,  q=0.07 MPa, was filled into the cylinder from the inflation hole. After the rubber film was deformed stably, the displacement of each measuring point on the film was measured by a laser displacement sensor, as shown in Figure 12. The measured experimental data and theoretical calculation results are shown in Table 2.

From Table 2, it can be seen that the experimental results are very close to the theoretical solution presented here, and the maximum error is 9.48%, which is much smaller than the allowable error measurement of 15%. Thus, it can be concluded that the theoretical solution given in this paper is reliable. Moreover, from Table 2, it can also be seen that the errors of the Hencky solution at multiple points are above 15%, which indicates that the Hencky solution is not an appropriate replacement.

## 5. Concluding Remarks

In this paper, the problem of axisymmetric deformation of the blistering circular thin polymer film under the action of uniformly distributed gas pressure was solved and its closed-form solution was presented by the power series method. The presented numerical example shows that the solution presented here was correct, and in blister test techniques, using the solution of a uniformly distributed transverse load as a substitute for the solution of uniformly distributed gas pressure will cause greater error. Generally, the error will increase with the increase in pressure. In addition, the related experiments of the blistering circular thin polymer film under uniformly distributed gas pressure were carried out, and the experimental results are compared with the theoretical solution. The comparison results show that the theoretical results are in good agreement with the experimental results, which ensures the reliability of the analytical solution given in this paper.

The work presented here should be of great significance to increase the accuracy of the blister test technique. In further studies, this work will be used for the derivation of measurement formulas of the blister test technique.

## Figures and Tables

**Table 1:** The deflection of the thin film when q is equal to different values.

*q*(MPa)	$w_{0.5a}\;(\mathrm{mm})$		$w_{0}\;(\mathrm{mm})$		$w_{0.5a}/w_{0}\;(\mathrm{mm})$		Error (%)
	A ^1^	B ^2^	A ^1^	B ^2^	A ^1^	B ^2^	
0.05	14.467	14.274	18.372	18.150	0.787	0.786	0.125
0.10	18.227	18.115	23.147	22.580	0.787	0.802	−1.878
0.15	20.865	20.503	26.497	25.293	0.787	0.810	−2.938
0.20	22.965	22.473	29.163	27.408	0.787	0.819	−4.125
0.50	31.168	31.398	39.581	37.516	0.787	0.836	−6.280
1.00	39.270	39.962	49.869	46.847	0.787	0.853	−8.326
1.50	44.953	46.958	57.086	54.063	0.787	0.868	−10.301

^1^ Denotes the Hencky solution and ^2^ denotes the solution presented here.

**Table 2:** Results in numerical values.

Measuring Points	Experiment Results (mm)	Theoretical Results (mm)		Error (%)	
		A ^1^	B ^2^	A ^1^	B ^2^
1	5.034	6.362	5.514	26.38	8.72
2	9.345	11.163	9.987	19.45	6.43
3	12.244	14.762	13.526	20.56	9.48
4	15.334	17.383	16.207	13.36	5.39
5	16.857	19.171	18.086	13.73	6.80
6	18.253	20.210	19.200	10.72	4.93
7	19.043	20.552	19.569	7.92	2.69
8	18.245	20.210	19.200	10.77	4.98
9	16.648	19.171	18.086	15.15	7.96
10	15.563	17.383	16.207	11.69	3.97
11	12.986	14.762	13.526	13.68	4.00
12	9.457	11.163	9.987	18.04	5.32
13	5.188	6.362	5.514	22.63	5.93

^1^ Denotes the Hencky solution and ^2^ denotes the solution presented here.
